# Regulation of Doublesex1 Expression for Environmental Sex Determination in the Cladoceran Crustacean Daphnia

**DOI:** 10.3389/fcell.2022.881255

**Published:** 2022-04-13

**Authors:** Yasuhiko Kato, Hajime Watanabe

**Affiliations:** Department of Biotechnology, Graduate School of Engineering, Osaka University, Suita, Japan

**Keywords:** *Doublesex1*, environmental sex determination, *Daphnia magna*, transcriptional regulation, post-transcriptional regulation, epigenetic regulation, male development

## Abstract

The cladoceran crustacean *Daphnia* produces only females by parthenogenesis in a healthy population. However, in response to environmental declines such as crowding and lack of foods, it produces eggs destined to become males that are genetically identical to females. During the development of the sexually committed eggs, DM domain-containing transcription factor *Doublesex1* (*Dsx1*) orchestrates male trait formation globally both in somatic and gonadal tissues. Recent studies have revealed that *Dsx1* expression is tightly controlled at transcriptional, post-transcriptional, and epigenetic levels to avoid sexual ambiguity. In this review, together with basic information on *Dsx1* structure and expression, we introduce the multi-layered *Dsx1* regulation and discuss how each regulation is interconnected for controlling male development in environmental sex-determining *Daphnia*.

## Introduction

Sex determination is a fundamental biological process that regulates differentiation not only of somatic tissues but also of gonads, resulting in the change of physiology and behavior in animals. It is broadly divided into two categories: genetic and environmental. Genetic sex determination is more spread in animals from invertebrates to vertebrates and relies on the difference of genetic elements between males and females (Bachtrog et al., 2014). In environmental sex determination, environmental cues trigger expression of the sex-determining gene and lead to any of male and female developmental programs. An environmental sex-determination system is also found in diverse taxa such as rotifers, nematodes, crustaceans, insects, fishes, and reptiles (Korpelainen, 1990). Exactly how the transient environmental cue initiates the sex-determining cascade and changes the developmental program has been of interest, but the regulatory mechanisms have remained unknown because the animals using the environmental sex-determination system have been poor genetic models.

**FIGURE 1:**
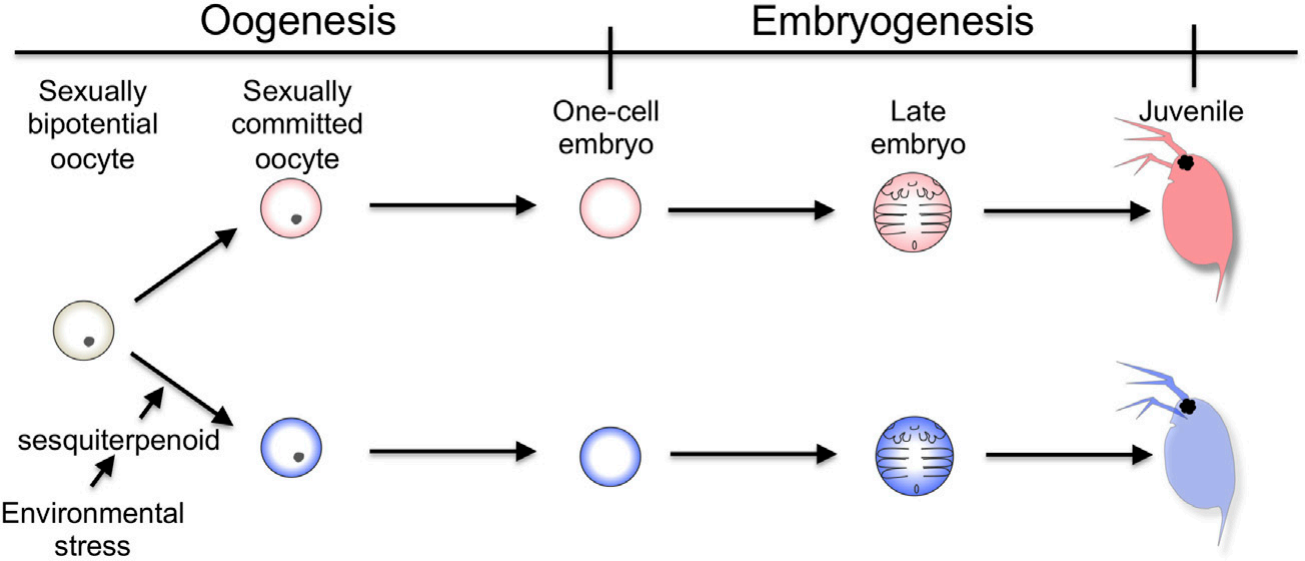
Timing of sexual commitment in environmental sex determination of *D. magna*. Under the healthy condition, the sexually bipotential oocyte is committed to be developed into a female. In contrast, by the mother, the information of environmental decline is detected and converted into sesquiterpenoid signaling, which commits the sexual fate of the oocyte to male.

The cladoceran crustacean is one of the models for studying environmental sex determination. In a healthy population, it produces females parthenogenetically. In contrast, when environmental quality declines, it also produces males that are genetically identical to females. The male then mates with a sexual female that also appears with environmental stressors, resulting in the generation of resting eggs that can withstand environmental hardships such as desiccation (Hebert, 1978). The commitment of sex to males occurs in the oocyte of the mother (Banta and Brown, 1929), meaning that the adult female senses the decline of the surrounding environment and transmits its environmental information to the next generation during oogenesis. Exposure of the crustacean sesquiterpenoid, methyl farnesoate, or its analog such as Fenoxycarb, also stimulates the production of male offspring (Olmstead and Leblanc, 2002; Tatarazako et al., 2003). This sesquiterpenoid-dependent commitment occurs at the late stages of oogenesis (Figure 1) (Olmstead and Leblanc, 2002; Kato et al., 2010). Taken together, in the cladoceran crustacean, the adult female senses the environmental cue and possibly converts it into sesquiterpenoid by the neuroendocrine system. Thereafter, this hormonal signaling induces sexual commitment of the oocytes to males, which in turn leads to the switching of the developmental program from the female-type to the male-type for male trait development.

*Doublesex* (*Dsx*) gene codes for the transcription factor harboring the DM domain and oligomerization domain and was firstly identified in *Drosophila melanogaster* as a sex determination gene (Hildreth, 1965). The DM domain is a DNA-binding domain that consists of an intertwined zinc-binding module followed by an alpha-helical recognition domain (Erdman and Burtis, 1993; Erdman et al., 1996). The oligomerization domain is known as the ubiquitin-associated domain and is required for dimerization that enhances the binding to the target sequence. *Dsx* gene is subjected to the sex-specific splicing, resulting in male- and female-specific isoforms (Baker and Wolfner, 1988; Burtis and Baker, 1989; Nagoshi et al., 1988). Each isoform shares the same DM domain but differs in the C-terminal oligomerization domain, which changes the interacting proteins and contributes to sex-specific gene regulation (Bayrer et al., 2005). In *Caenorhabditis elegans*, the male regulator *mab-3* gene was found to have the DM domain (Shen and Hodgkin, 1988). The orthologous genes have been further identified in vertebrates as “Doublesex- and mab-3 regulated transcription factor (DMRT)” and have been proved to function as a sexual regulator (Raymond et al., 2000; Matsuda et al., 2002; Ge et al., 2017; Smith et al., 2009). In contrast to deep conservation of this gene family for sex-related development, the regulators that act upstream of the DMRT genes in the sex-determining cascade are diverse in animals (Wilkins, 1995; Herpin and Schartl, 2015).

Here we review the characterization and regulation of *Dsx* ortholog named *Doublesex1* (*Dsx1*) in the cladoceran crustacean *Daphnia magna*. We will introduce the functional conservation of *Dsx1* in sexual development and describe how this ancestral sexual regulator is controlled by environmental cues and during embryogenesis in *D. magna*.

### *Dsx1* Lacks Sex-specific Splicing Variants

**FIGURE 2:**
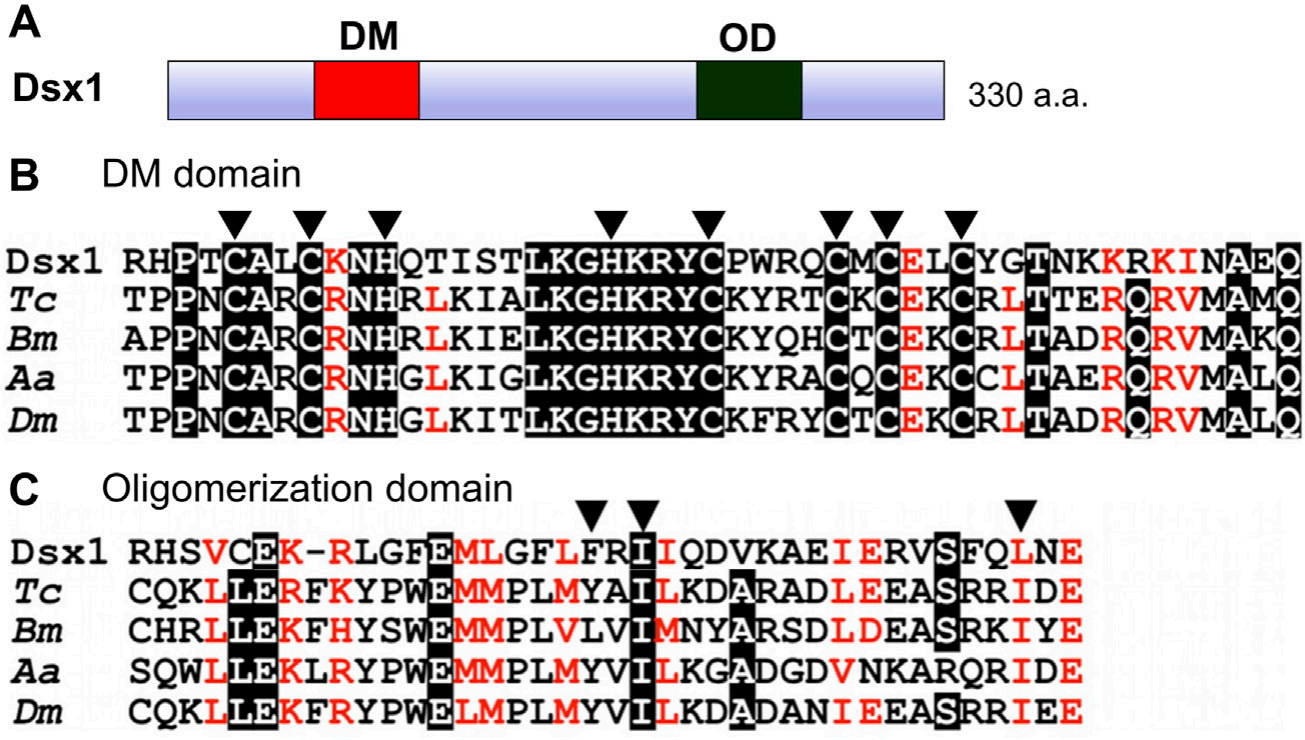
Domain organization of *Dsx1*. **(A)** Schematic representation of *Dsx1*. DM: DM domain, OD: Oligomerization domain. **(B)** Amino acid sequence alignment of DM domains from arthropod *Dsx* orthologs. Arrowheads indicate zinc-chelating amino acids. **(C)** Amino acid sequence alignment of oligomerization domains of arthropod *Dsx* orthologs. Arrowheads indicate three non-polar amino acids involved in the formation of the interface. *Dsx1*, *D. magna Dsx1*; Tc, *Tribolium castaneum*; Bm, *Bombyx mori*; Aa, *Aedes aegypti*; Dm, *Drosophila melanogaster*. Adapted and modified from Kato et al. (2011).

*D. magna* has two *Dsx* orthologs, *Dsx1* and *Dsx2* (Kato et al., 2011). Because *Dsx2* seems to have lost sex-related function (Kato et al., 2011), its characterization and potential function is not focused in this review. *D. magna Dsx1* has the DM domain and oligomerization domain at the N-terminus and C-terminus respectively like *Drosophila* and the other insect *Dsx* proteins (Kato et al., 2011) (Figure 2A). The amino acid sequence of *Dsx1* DM domain is well-conserved including zinc-chelating amino acids (Figure 2B). The oligomerization domain shows a more divergent sequence, but three non-polar amino acids involved in the formation of the interface are conserved (Figure 2C), suggesting that modes of transcriptional regulation of the *D. magna Dsx1* are similar to those of *Drosophila Dsx*.

**FIGURE 3:**
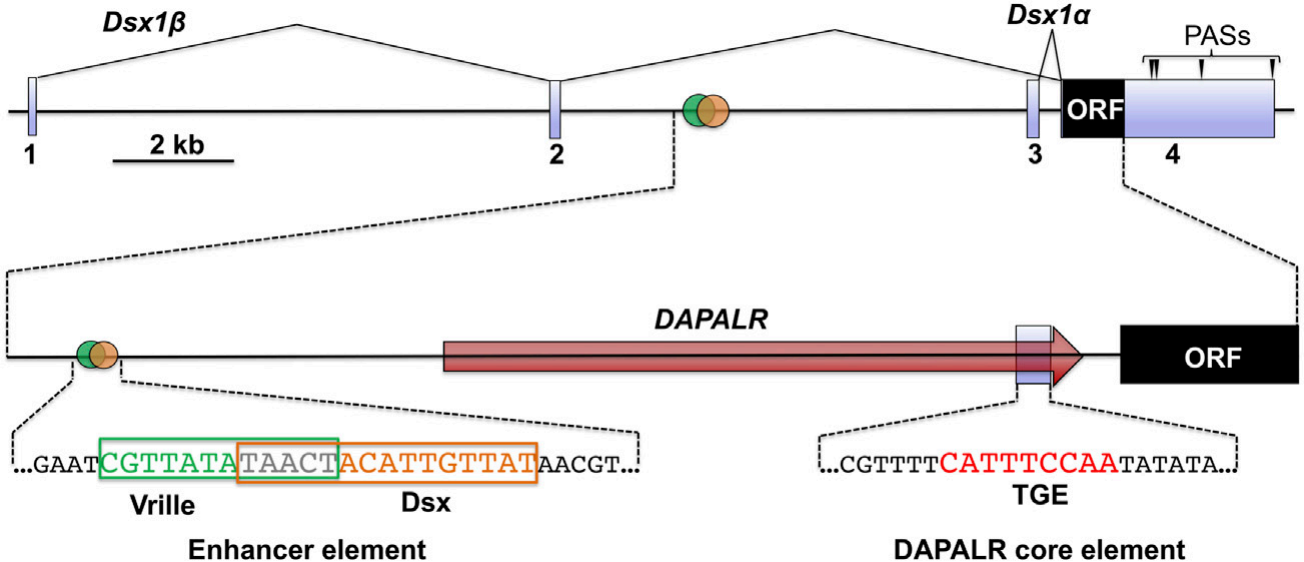
Genomic structure of *Dsx1* gene. Exons of *Dsx1* are indicated with light blue boxes. The red arrow shows the region and direction of *DAPALR*. Polyadenylation sites are indicated with arrowheads. The enhancer element that includes the potential Vrille and *Dsx* binding sites is shown with its sequence. The position of the *DAPALR* core element is shown with the sequence that is similar to the TRA-2/GLI element (TGE). Adapted and modified from Kato et al. (2018).

The *D. magna Dsx1* gene is approximately 20 kb in length and consists of four exons (Kato et al., 2011) (Figure 3). The first and second introns are approximately 9 and 8 kb, each of which is considerably longer than the average size of introns (392 bp) on the *D. magna* genome, which may imply that important regulatory sequences are in the introns. Two transcripts, *Dsx1α* and *Dsx1β*, are produced from the *Dsx1* locus by alternative promoter usage: *Dsx1α* utilizes exon 3 and exon 4, and *Dsx1β* is encoded in exons 1, 2, and 4. The *Dsx1* ORF is included in exon 4 and is not spliced in a sex-specific manner, demonstrating that, unlike *Drosophila* and other insects’ *Dsx*, the same polypeptide is produced in both sexes. The 3′ UTR region contains four alternative polyadenylation sites, resulting in the synthesis of mRNAs with different 3′ UTR lengths (Kato et al., 2011). Even though *Dsx1* gene is not regulated by sex-specific splicing, these sequence characteristics suggest that this gene is intricately regulated at transcriptional and post-transcriptional levels.

### Male-Specific Expression of *Dsx1*

**FIGURE 4:**
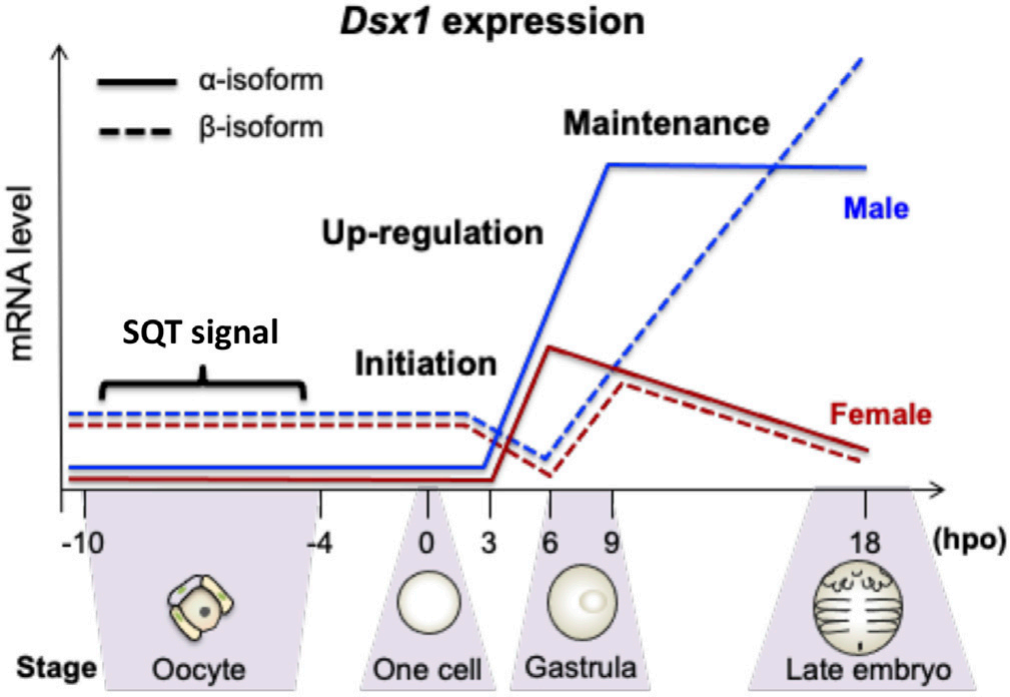
Temporal expression patterns of *Dsx1* isoforms during late oogenesis and early embryogenesis. hpo, hour post ovulation; SQT, sesquiterpenoid. Adapted from Mohamad Ishak et al. (2017).

Temporal *Dsx1* expression is divided into three phases: initiation, upregulation, and maintenance (Figure 4) (Mohamad Ishak et al., 2017). Before 6-hour post ovulation (hpo), *Dsx1α* mRNA is synthesized both in males and females (initiation phase). Around the timing of gastrulation, from 6 to 9 hpo, its expression is upregulated only in males (upregulation phase). In contrast to *Dsx1α* mRNA, *Dsx1β* mRNA is deposited as a maternal RNA in both sexes. After consumption of maternal RNAs, zygotic *Dsx1β* expression begins in both sexes and its male-specific upregulation occurs at 3 h later than the timing of *Dsx1α* upregulation. This difference of timing for male-specific upregulation of each isoform suggests that maternal sesquiterpenoid signaling first influences the *Dsx1α* promoter Thereafter, in males, expression of both isoforms is steadily maintained. *Dsx1α* is further upregulated at the instar 5 when the secondary sex characteristics appear. In females, the *Dsx1* locus is persistently silenced except for synthesis of the maternal *Dsx1β* mRNA (Nong et al., 2017).

**FIGURE 5:**
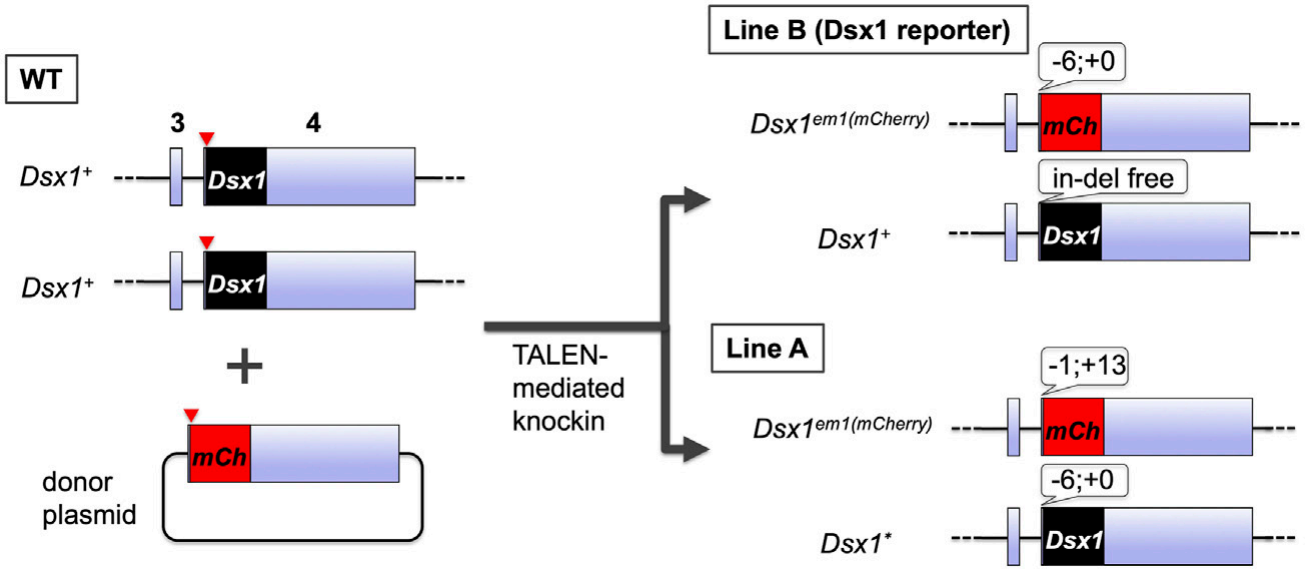
Generation and genotypes of the transgenic *D. magna* harboring the *mCherry* coding sequence at the *Dsx1* locus. The TALANs induced a double-strand break at the start codon of *Dsx1* ORF on exon 4. Concomitantly, it also cleaved near the start codon of *mCherry* ORF fused with *Dsx1* 3′ UTR on the donor plasmid, resulting in the generation of two transgenic lines, Line A and Line B.

To investigate where the *Dsx1* activation occurs throughout development, the transgenic *D. magna* for visualizing the *Dsx1* expression was generated (Nong et al., 2017). TALENs that introduce double-strand break at the start codon of *Dsx1* in its exon 4 was constructed and used for non-homologous end-joining mediated knock-in of the donor plasmid harboring the mCherry ORF and *Dsx1* 3′ UTR (Figure 5). Coinjection of the TALEN mRNAs with the donor plasmid led to the establishment of two transgenic lines (Line A and Line B) showing mCherry fluorescence. Both lines have a single copy of the mCherry reporter on one of the *Dsx1* alleles. In Line A, another *Dsx1* allele is mutated with a 6-bp deletion at the start codon, which suggests that this line does not produce any intact *Dsx1* protein (Nong et al., 2020). In contrast, Line B has a wild-type *Dsx1* gene on another allele. This genotype enables Line B males to develop sex-specific morphology such as elongation of first antennae and to have the reproductive ability for producing sexual eggs by copulating with females, even though those male-specific traits are slightly feminized (Nong et al., 2017). Localization of mCherry fluorescence was consistent with *Dsx1* mRNA localization that was analyzed by *in situ* hybridization (Kato et al., 2011). Therefore, Line B has been used as a *Dsx1* reporter line that recapitulates *Dsx1* expression (Mohamad Ishak et al., 2017; Kato et al., 2018; Perez et al., 2021).

**FIGURE 6:**
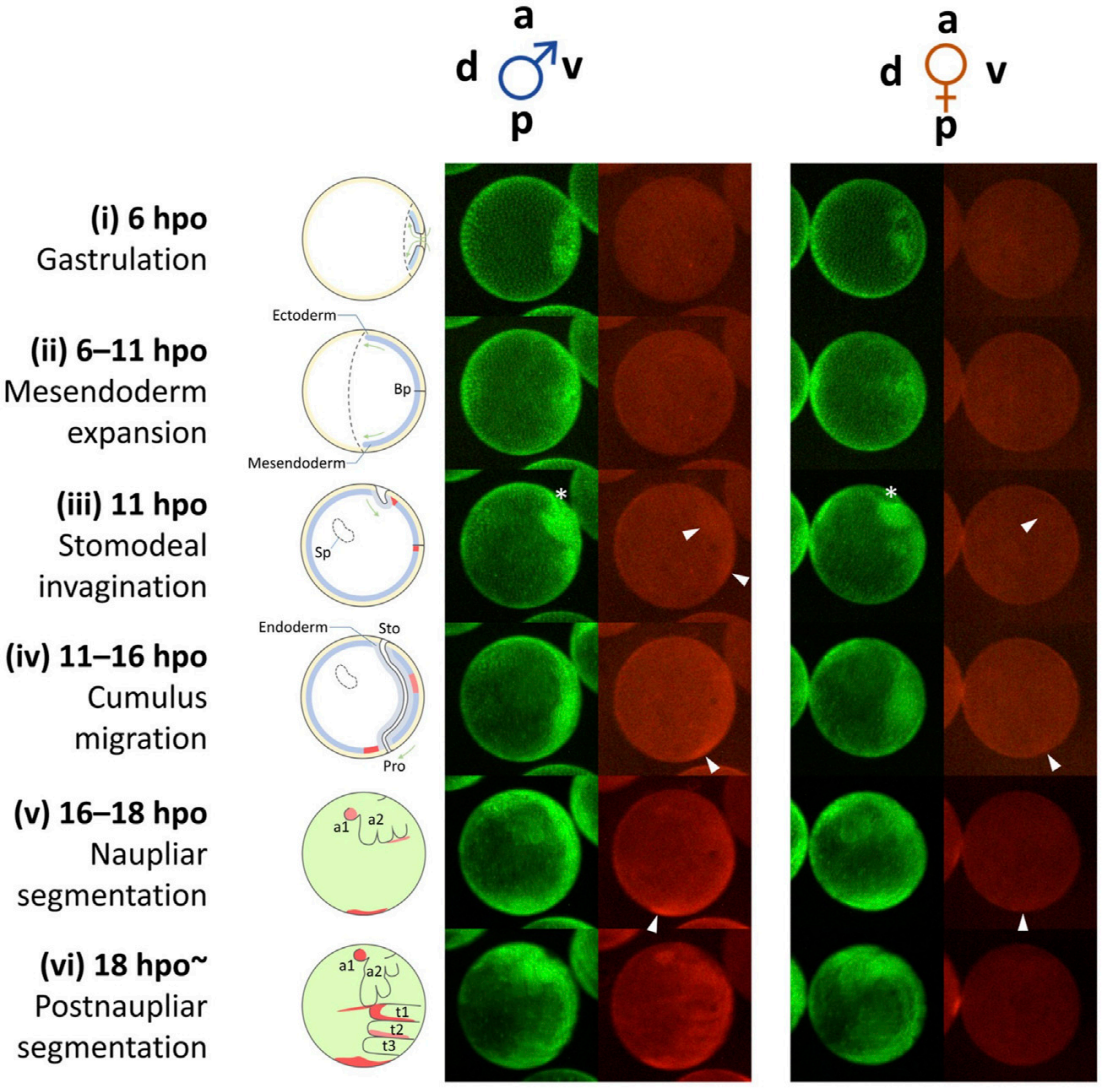
Dynamic progression of *Dsx1*-expressing cells during early embryogenesis from gastrulation until thoracic segmentation. Schematic diagrams on the left illustrate the simple anatomy of the embryos at the corresponding time points. The diagrams from stages (i) to (iv) show images of embryonic sections. The diagrams (v) and (vi) show superficial views of embryos. a, anterior; p, posterior; v, ventral; d, dorsal; Bp, blastopore; Sp, Scheitelplatten; Sto, stomodeum; Pro, proctodeum; t1–3, thoracic segments; a1, first antenna; a2, second antenna; hpo, hours post ovulation. White arrowheads indicate mCherry-expressing cell clusters that migrate in an anterior-to-posterior direction. An asterisk indicates the site of invagination. Adapted from Nong et al. (2017).

Live imaging of mCherry fluorescence in Line B male embryos revealed the dynamic change of spatial expression patterns in early embryos (Nong et al., 2020) (Figure 6). During the initiation phase of *Dsx1*, no clear mCherry signal was detected probably due to low *Dsx1* expression levels (Figure 6, stage i and ii). In the upregulation phase, two mCherry signals were detected at 11 hpo. One was the cell cumulus appearing around the invagination, and the other was around the blastopore (Figure 6, stage iii). The mCherry-expressing cumulus moved to the vicinity of the blastopore, and the signal appeared to weaken gradually (Figure 6, stage iv). In parallel, another mCherry-expressing population migrated from the blastopore to the posterior region (Figure 6, stage iv). When naupliar segmentation occurred, the population of cells showing strong mCherry fluorescence converged in the posterior growth zone although it is not clear whether the two mCherry-expressing cell populations merged (Figure 6, stage v). In other animals, cell populations showing similar migration behavior are known as a primary organizer that induces differentiation in the adjacent area (Bénazéraf and Pourquié, 2013; Akiyama-Oda and Oda, 2003). This suggests that the migrating cell population may induce sexual differentiation in their surrounding cells. The posterior growth zone is known to supply the progenitor cells for the formation of thoracic appendages (Martin and Kimelman, 2009), suggesting that, in male embryos of *D. magna*, this region distributes masculinized progenitor cells for the development of the male-specific structure of thoracic appendages. Strong mCherry fluorescence was also observed in the buds of the first antennae, and in parallel, strong mCherry fluorescence was detected at the boundary between the head and thoracic segments (Figure 6, stage v). Later, at 18 h, more intense mCherry fluorescence was detected in the first thoracic appendage (Figure 6, stage vi), where hooks for female capture are formed at the later juvenile stage. During these earlier embryonic stages, rather broader mCherry fluorescence patterns were observed, indicating that the embryos are still sexually immature. Localization of mCherry fluorescence of female embryos was similar but its intensity was weaker compared to male embryos until the naupliar segmentation stage. Thereafter, there was no mCherry fluorescence in female embryos (Figure 6).

**FIGURE 7:**
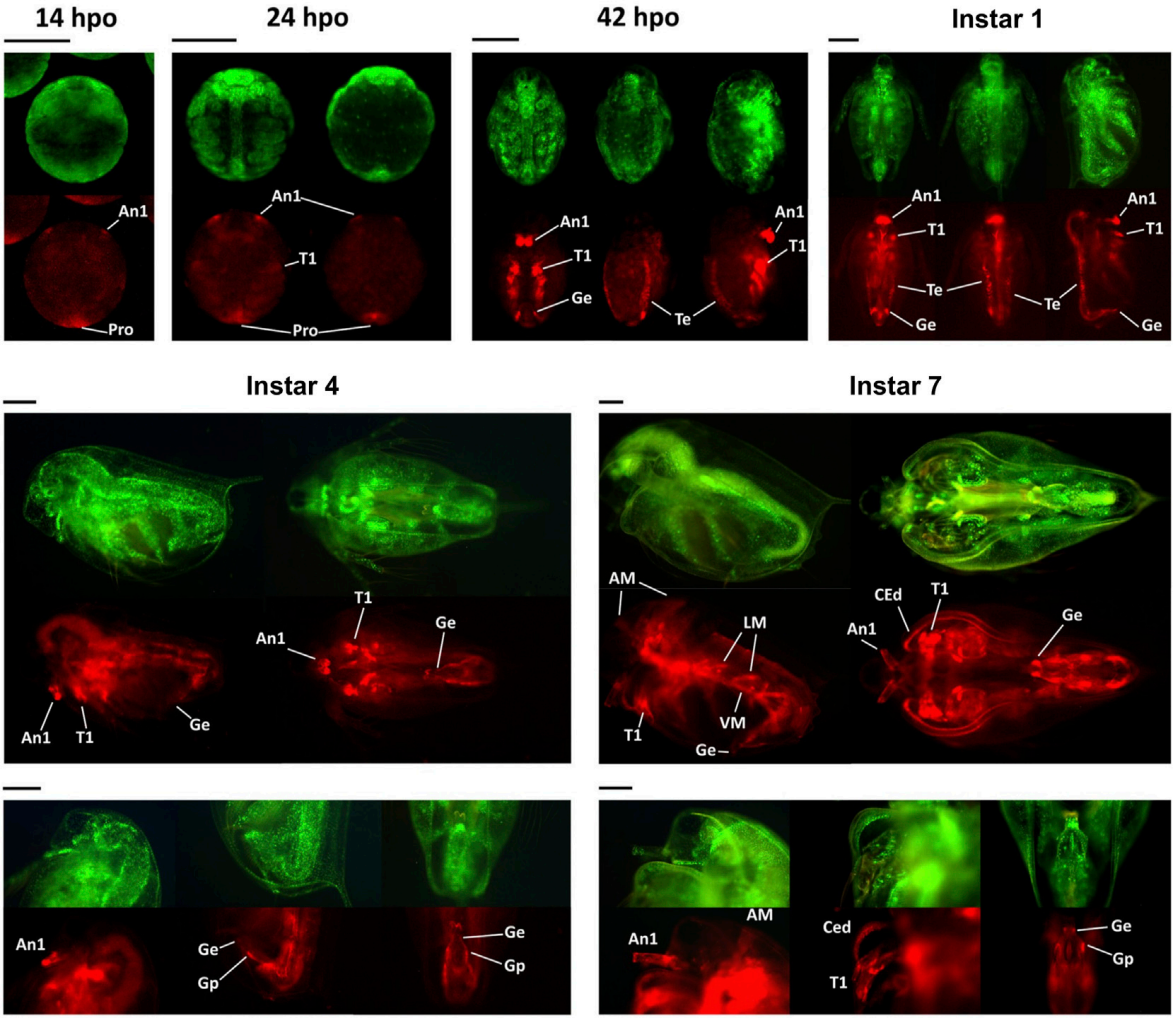
Spatially and temporally specific expressions of *mCherry* in the *Dsx1* reporter strain. All embryos and daphniids shown in this figure are males of the Line B. hpo, hours post-ovulation; dpo, days post-ovulation. For the 14 hpo panel: ventral view. For the 24 hpo panel: ventral view (left) and dorsal view (right). For the 42 hpo and instar 1 panels: ventral view (left), dorsal view (middle), and side view with the ventral side facing right (right). A1, first antenna; T1, first thoracic leg; Pro, proctodeum; Ge, genital (i.e., penis in this case); Te, testis; AM, antennal muscle; LM, lateral muscle; VM, ventral muscle; CEd, carapace edge; Gp, gonopore. Scale bars = 200 μm. Adapted from Nong et al. (2017).

As embryonic development progressed toward the first instar stage that corresponded to 72 hpo, mCherry signal was localized to organs characteristic of males, first antennae, first thoracic leg, external genitalia, and testis (Figure 7, instar 1). The expression of *Dsx1* in these male-specific traits stably continued until the fourth instar stage (Figure 7, instar 4). After males showed secondary sex characteristics at the instar 5, mCherry fluorescence expanded to the other several male-specific structures, including the carapace edge below the helmet, the tip of the penis, and the skeletal muscle (Figure 7, instar 7) (Nong et al., 2020). These imaging results suggest that *Dsx1* is tightly regulated by time and place for controlling male development.

### *Dsx1* is Essential for Male Trait Development

**FIGURE 8:**
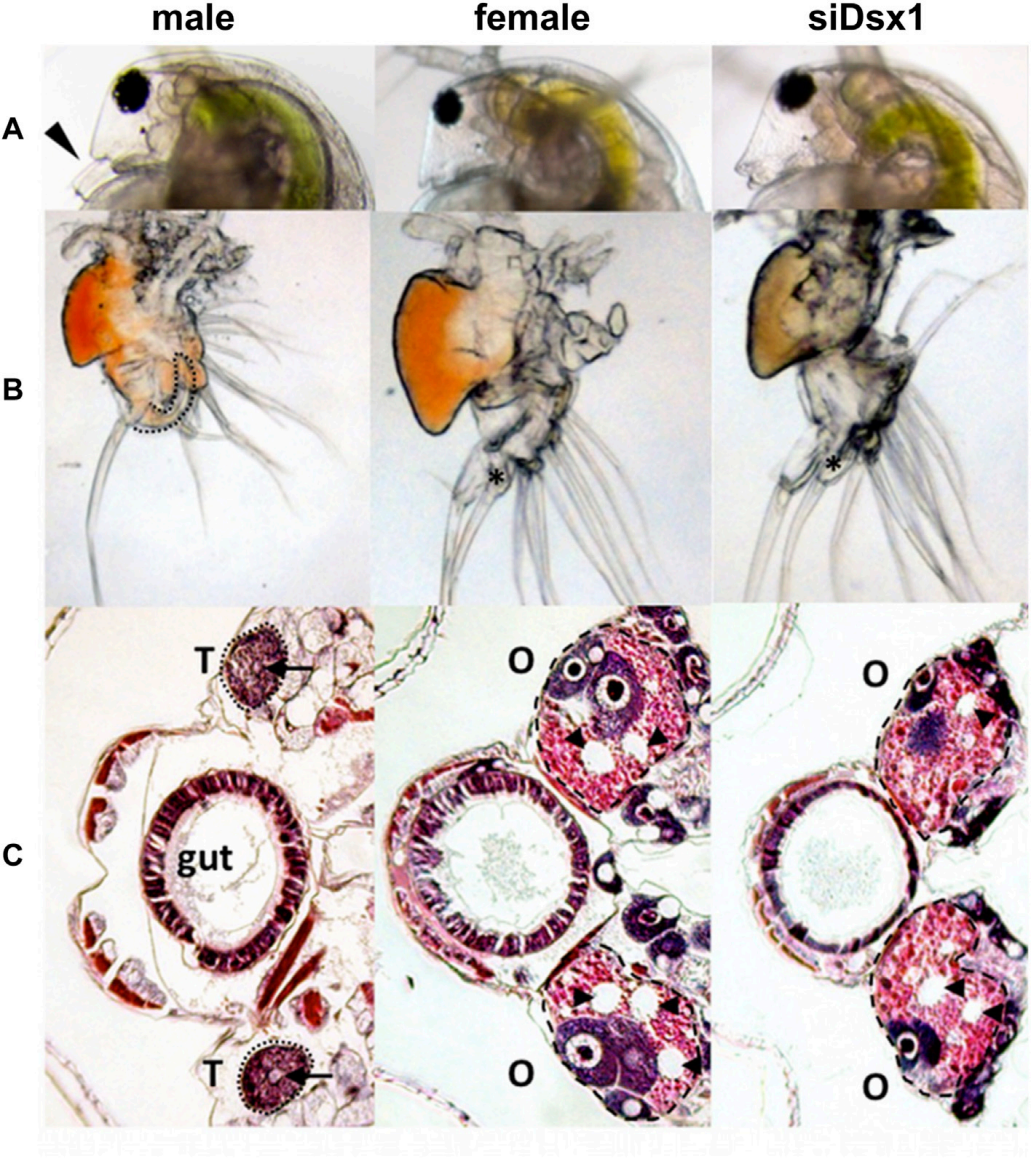
Dimorphic development of *D. magna*. Eggs induced to become males were obtained from *D. magna*. After injection of the synthesized dsRNA, sexually dimorphic phenotypes were examined at the fifth or sixth instar except for the first antennae (third instar). The first two columns represent normal male and female phenotypes, respectively. The third and fourth columns represent phenotypes of individuals injected with dsRNAs of *dsx1* and *dsx2*, respectively. **(A)** Lateral view of the head. Arrowheads indicate the first antennae. **(B)** First thoracic limb. The dotted line shows the outline of the stout chitinized hook. A female-type long filament corresponding to the hook is labeled with an asterisk. **(C)**: Gonad. Daphnids were embedded in paraffin and sectioned, followed by standard hematoxylin and eosin staining. Dorsal is left, ventral is right. Dotted circled lines show gonads at both sides of a gut. T and O indicate testis and ovary, respectively. Arrowheads indicate large lipid droplets lying among the eosinophilic yolk granules. Arrows indicate lumens into which the mature spermatozoa are released. Adapted from Kato et al. (2011).

A previous study reported that knockdown of *Dsx1* led to feminization in males (Kato et al., 2011). Long dsRNA targeting *Dsx1* mRNA was injected into eggs that were destined to be developed into males. By *Dsx1* RNAi, the first antennae of males became short like those of females (Figure 8A) and a copulatory hook for capturing females was lost from the first thoracic leg (Figure 8B). In addition to the feminization of somatic tissues, the gonads also showed sex reversal from testes to ovaries (Figure 8C). When the chemically modified *Dsx1*-targeting siRNA was injected, the effects of *Dsx1* silencing in adults became severe enough to allow the knockdown males for laying eggs (Kato et al., 2018), showing that *Dsx1* is the key sex determination factor in *D. magna*.

**FIGURE 9:**
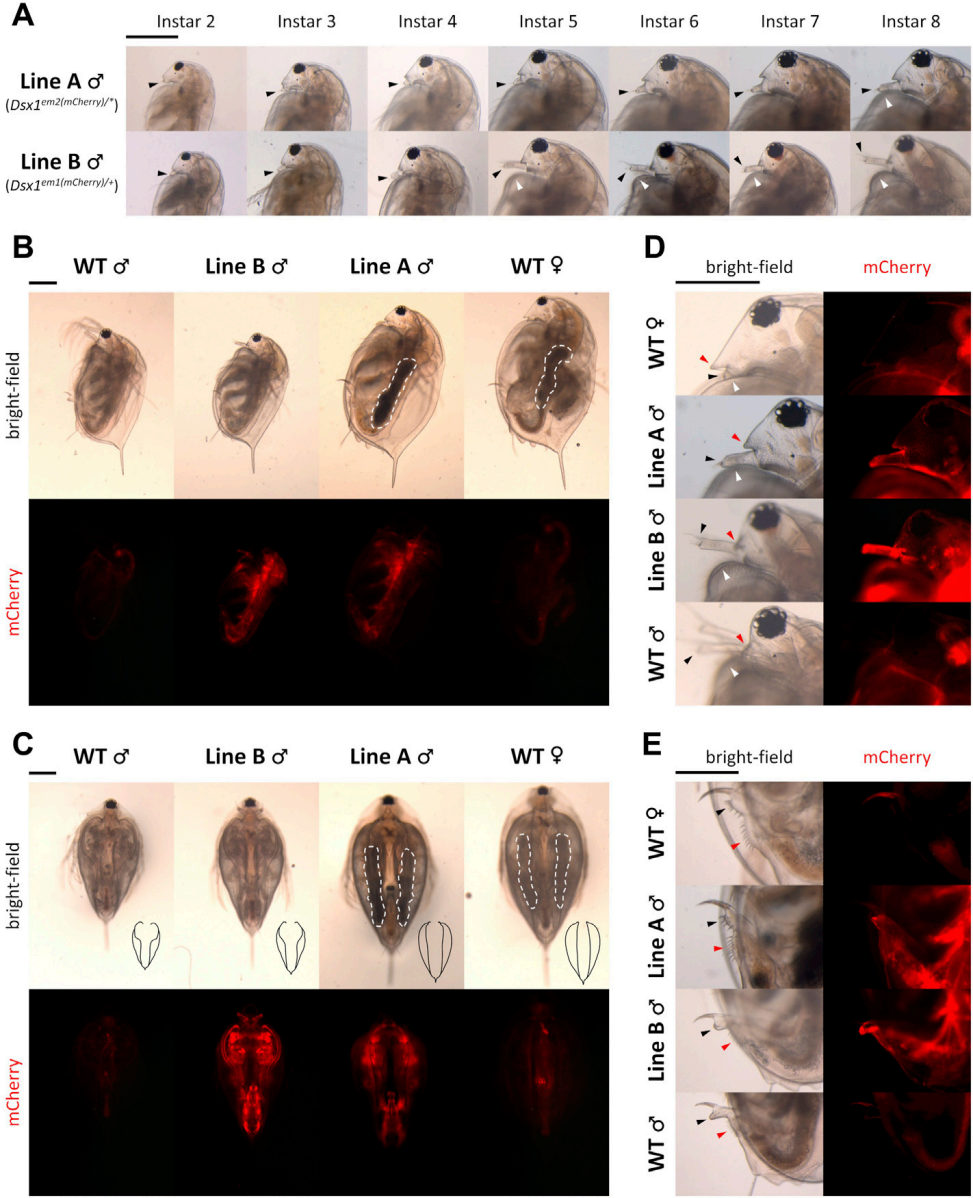
Feminization in the phenotype of *Dsx1* mutants. Head region with a focus on the first pair of antennae of Line A males and Line B males of various ages. Until instar 4, juvenile stages of daphniids continue. Transition to fully mature adults usually occurs when the animal reaches instar 5, which is approximately 1 week after birth. Black-arrowheads: first antennae. White arrowheads: carapace curve that reveals copulation hooks in males. **(B)** Full body, side view of instar 8 daphniids. White dotted lines indicate ovary-like gonads, which appeared darker because of the accumulation of yolk protein. **(C)** Full body, ventral view of instar 8 daphniids. Black drawings at the bottom right corner of each bright-field photo are tracing lines scaled down to a 1:2.26 ratio depicting the outline of the carapace edge. White dotted lines have the same meaning as in panel **(B)**. **(D)** Head region, side view of instar 8 daphniids. Black and white arrowheads have the same meaning as in panel **(A)**. Red arrowheads indicate the rostrum. **(E)** Genital and anal region, side view of instar 8 daphniids. Black arrowheads: genital. Red arrowheads: anus. Each panel from **(A–E)** has one scale bar indicating 0.5 mm, which is shared among all photos of the same panel. Adapted from Nong et al. (2020).

Phenotyping of the *Dsx1* mutants Line A and Line B aforementioned also showed *Dsx1* dependency of male trait development (Figure 4) (Nong et al., 2017; Nong et al., 2020). Compared to Line B males, Line A males showed more severe feminization throughout development, which were obvious in the first antennae and carapace (Figure 9A). The first antennae of Line A were as short as those of wild-type females in younger juveniles in contrast to Line B males with rather elongated first antennae. At instar 5 when the secondary sex characteristics appeared, the difference in the degree of feminization between the two mutants became clearer. Line B male showed the transformation of first antennae and carapace into the mature adult-like structures at this instar whereas Line A gradually transformed morphology of those two male traits across the following instar stages. Feminization of Line A males was also observed throughout the body such as larger body size (Figures 9B,C), the modest opening of the ventral carapace (Figure 9C), bulky head (Figure 9D), female-like genital and anus (Figure 9E). In addition, Line A male developed ovary-like darkened gonads but did not produce any eggs (Figures 9B,C). Male-specific traits are developed exclusively dependent on *Dsx1* activity in *D. magna*.

## Transcriptional, Post-Transcriptional, Epigenetic Control of *Dsx1*

### bZip Transcription Factor Vrille Upregulates *Dsx1* Expression

**FIGURE 10:**
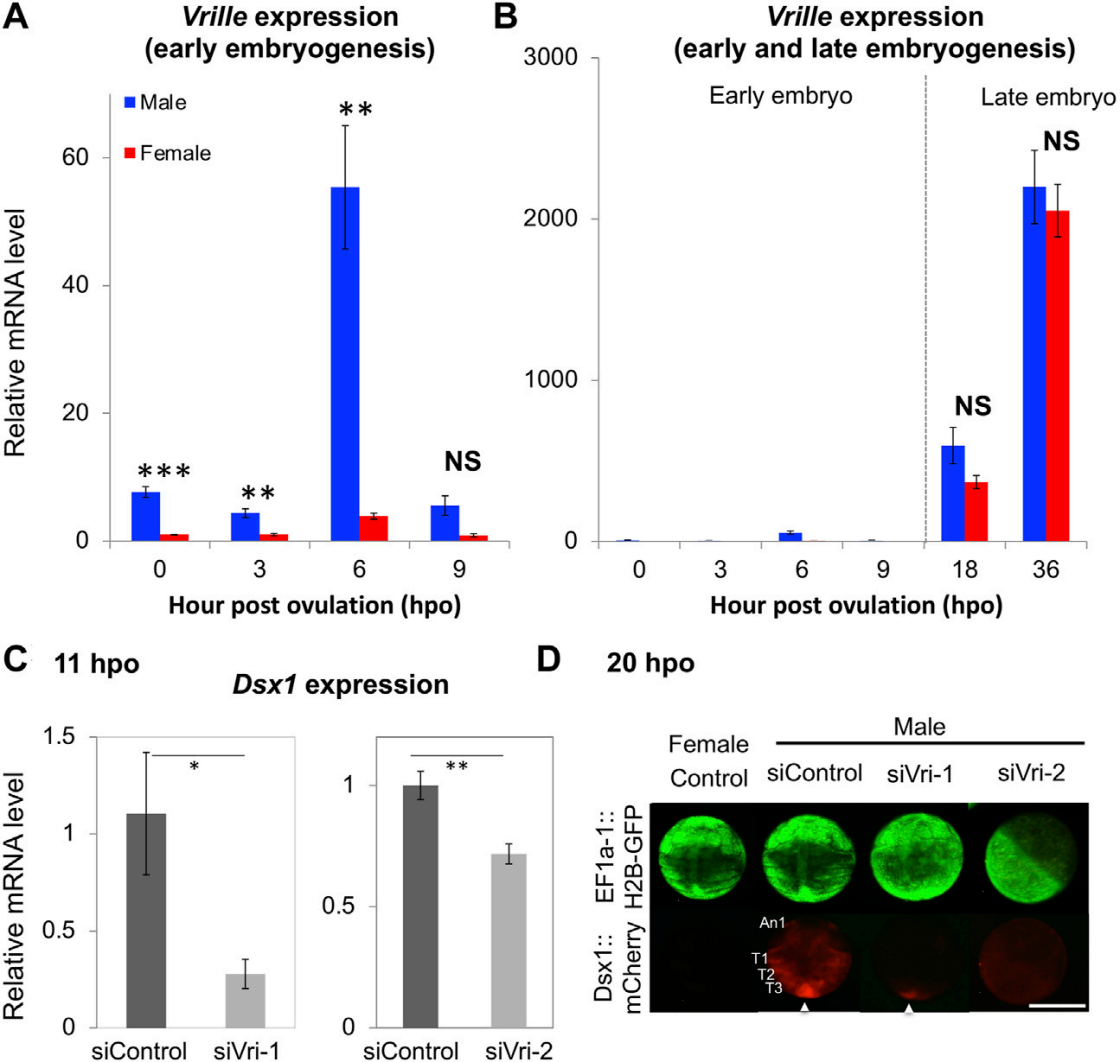
*Vrille* (*Vri*) expression in wild-type and knockdown embryos of *D. magna*. **(A)** Sexual dimorphism of *Vri* expression at early embryonic stages (0, 3, 6, and 9-hpo) of wild-type. **(B)** Non-sex-specific *Vri* expression in the late embryos (18 and 36-hpo) of wild-type. The *Vri* expression levels were normalized to the reference gene expression levels (ribosomal protein *L32*). The normalized *Vri* expression in female embryos at 0-hpo was set to one. **(C)** The *Dsx1* expression level at 11 h after siRNA injection was measured by qRT-PCR. siControl, siVri-1 and siVri-2 indicate control-, Vri_siRNA_1, and Vri_siRNA_2 injected males, respectively. Each experiment was performed in biological triplicate. For each replicate, nine or ten embryos were pooled in one tube, subjected to total RNA extraction, and used for quantitation. **(D)** Spatial expression pattern of *mCherry* as a reporter of *Dsx1* gene expression in *Vrille* knockdown embryos at 20-hpo. An1, first antennae; T1, thoracic appendage 1; T2, thoracic appendage 2; T3, thoracic appendage 3; An arrowhead indicates the posterior growth zone; Scale bars: 200 μm. (Student’s *t*-test; *p < 0.05; **p < 0.01; ***p < 0.001; NS, No significant different). Adapted from Mohamad Ishak et al. (2017).

On the *D. magna* genome, upstream of the *Dsx1α* transcription start site, there is an element that resembles the consensus sequence of the binding site for the bZip transcription factor Vrille. This element overlaps a potential *Dsx* binding site (Figure 3) (Mohamad Ishak et al., 2017). A similar element harboring both the bZip transcription factor and *Dsx* binding sites had been characterized well as an enhancer of yolk protein gene in *Drosophila* (An and Wensink, 1995). Importantly, in *D. magna*, male-specific expression of *Vrille* occurred at 6 hpo before upregulation of *Dsx1* begins (Figure 10A) even though *Vrille* did not show any sexually dimorphic expression in the later stages of embryos (Figure 10B). Vrille knockdown reduced *Dsx1* expression in male embryos (Figures 10C,D) whereas its overexpression in females activated *Dsx1* expression and in turn led to the development of male trait, elongation of first antennae (Mohamad Ishak et al., 2017). Disruption of the Vrille binding site on the *Dsx1α* promoter region inhibited *Dsx1* activation in male embryos, demonstrating that Vrille is required for *Dsx1* activation during the upregulation phase. Interestingly, there was a potential binding site of the sesquiterpenoid receptor Methoprene-tolerant (Met) in the *Vrille* promoter, suggesting the possibility that the Met may directly regulate *Vrille* expression (Mohamad Ishak et al., 2017). In *Drosophila*, *Vrille* functions as a sex regulatory factor downstream of *Dsx* (Lebo et al., 2009). *Vrille* might be repeatedly co-opted in the sex-determining pathways with the different hierarchical position in animals as reported in the other sex-determining genes (Herpin and Schartl, 2015).

### Sex Differences of Histone Modification and DNA Methylation Level on *Dsx1* Locus

A comprehensive epigenomic analysis of *Daphnia pulex* revealed that, on the *Dsx1* locus, there were sex differences of active and repressive histone marks, histone H3 trimethylation at lysine 4 (H3K4me3), and histone H3 trimethylation at lysine 27 (H3K27me3) in addition DNA methylation status (Kvist et al., 2020). The H3K4me3 level was around 300-fold higher but the H3K27me3 level was around 5,000-fold lower in males compared to those in females. CpG methylation level was higher in females. These epigenetic marks suggest opened and closed chromatin states in males and females respectively. Although epigenetic marks on *D. magna Dsx1* remain unknown, a previous study found that *Dsx1* enhancer element-targeting Crispr/Cas9 could not induce any mutation on the *Dsx1* enhancer element in female embryos but resulted in indel formation in males (Mohamad Ishak et al., 2017). CRISPR/Cas9 is known to have weaker activity against the closed chromatin (Chen et al., 2016; Daer et al., 2016; Jensen et al., 2017), supporting the sex difference of the epigenetic state on the *Dsx1* locus.

### LncRNA DAPALR Antagonizes Shep-Dependent Translational Repression of *Dsx1*

**FIGURE 11:**
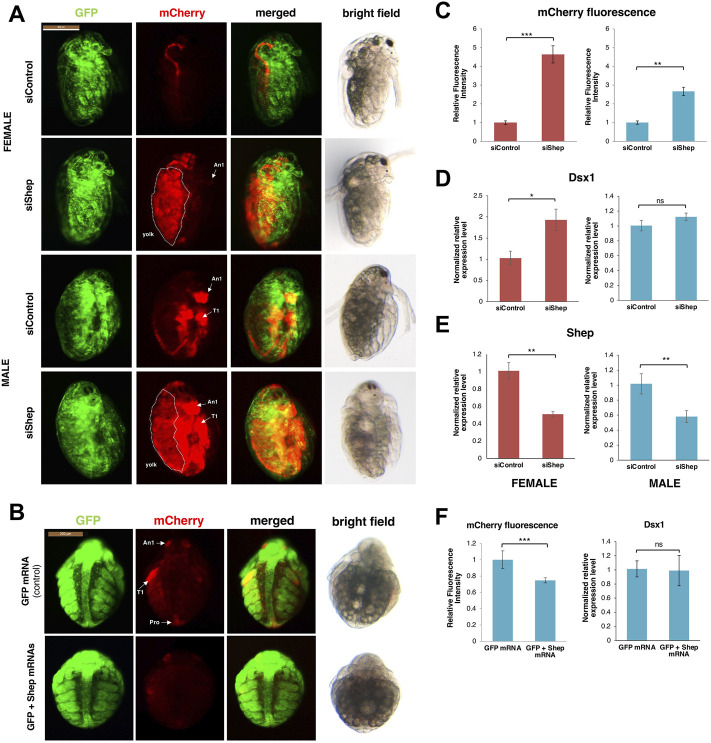
*Shep* loss of function analysis. **(A)** Lateral view of female and male embryos of *Dsx1* reporter strain injected with control siRNA and *Shep* siRNA and observed at 48 h after injection. mCherry fluorescence allowed visualization of *Dsx1* expression while GFP fluorescence in the nucleus enabled observation of body structures. The merged images of mCherry and GFP and the bright field images were used to understand the localization pattern of *mCherry* expression. An1: first antennae, T1: first thoracic leg, dotted lines: yolk area. **(B)** Ventral view of male embryos of *Dsx1* reporter strain injected with *GFP* mRNA as control and *GFP* plus *Shep* mRNA observed at 30 h after injection. mCherry fluorescence allowed visualization of *Dsx1* expression while GFP fluorescence in the nucleus enabled observation of body structures. The merged images of mCherry and GFP and the bright field images were used to understand the localization pattern of mCherry expression. An1: first antennae, T1: first thoracic legs, Ge: genital. **(C)** Relative mCherry fluorescence intensity calculated between *Shep* siRNA- and control siRNA-injected female (red) and male (blue) embryos. Error bars indicate the standard error of the mean (*n* = 5). **(D)** Gene expression profile of *Dsx1* in control siRNA- and *Shep* siRNA-injected female (red) and male (blue) embryos. **(E)** Gene expression profile of *Shep* in control siRNA- and *Shep* siRNA-injected female (red) and male (blue) embryos. **(F)** Relative mCherry fluorescence intensity calculated between *GFP* mRNA- and *GFP* plus *Shep* mRNA-injected male embryos (left figure). Error bars indicate the standard error of the mean (*n* = 5). Gene expression profile of *Dsx1* in *GFP* mRNA- and *GFP* plus *Shep* mRNA-injected male embryos (right figure). RT-qPCR results are shown as expression levels normalized with housekeeping genes *L32*, *L8*, and *Cyclophilin* and relatively compared to the control. Error bars indicate the standard error of the mean (*n* = 3). *p < 0.05, **p < 0.01, ***p < 0.001, ns: not significant (Student’s *t*-test). Adapted from Perez et al. (2021).

The RNA binding protein Alan shepard (Shep) ortholog was proved as a post-transcriptional regulator for *Dsx1α* mRNA in *D. magna* (Perez et al., 2021). The relationship between *Shep* and *Dsx1α* mRNA was found unexpectedly based on phenotypic analysis of female embryos injected with *DsRed2* reporter RNA harboring the *Dsx1α* or *Dsx1β* 5′ UTR (Kato et al., 2018). Despite lacking the *Dsx1* ORF, the chimeric *DsRed2* RNA including the *Dsx1α* 5′ UTR induced development of the male trait and activation of *Dsx1* in females. The RNA that coded for only *Dsx1α* 5′ UTR also led to *Dsx1* activation. To investigate the molecular mechanism underlying the unexpected function of *Dsx1α* 5′ UTR for *Dsx1* regulation, the proteins interacting with *Dsx1α* 5′ UTR were examined by the MS analysis with *D. magna* lysates and one of the identified proteins was Shep. *D. magna Shep* gene is expressed both in males and females during embryogenesis. Silencing of *Shep* in the *Dsx1* reporter line increased *mCherry* expression in male and female embryos (Figures 11A,C). In contrast, overexpression of *Shep* reduced the *mCherry* expression in the *Dsx1* reporter line (Figures 11B,F). However, both loss- and gain-of-function did not change *Dsx1* transcript levels (Figures 11D–F), suggesting that Shep represses *Dsx1* expression at a post-transcriptional level. *Dsx1α* 5′ UTR has a sequence like “tra-2 and GLI element (TGE)” (Figure 3), which has been identified as a binding site of Sup-26, an ortholog of Shep in *C. elegans*. *In vitro* reporter assay demonstrated that Shep represses translation of *Dsx1α* mRNA via TGE (Figure 13A) (Perez et al., 2021).

**FIGURE 12:**
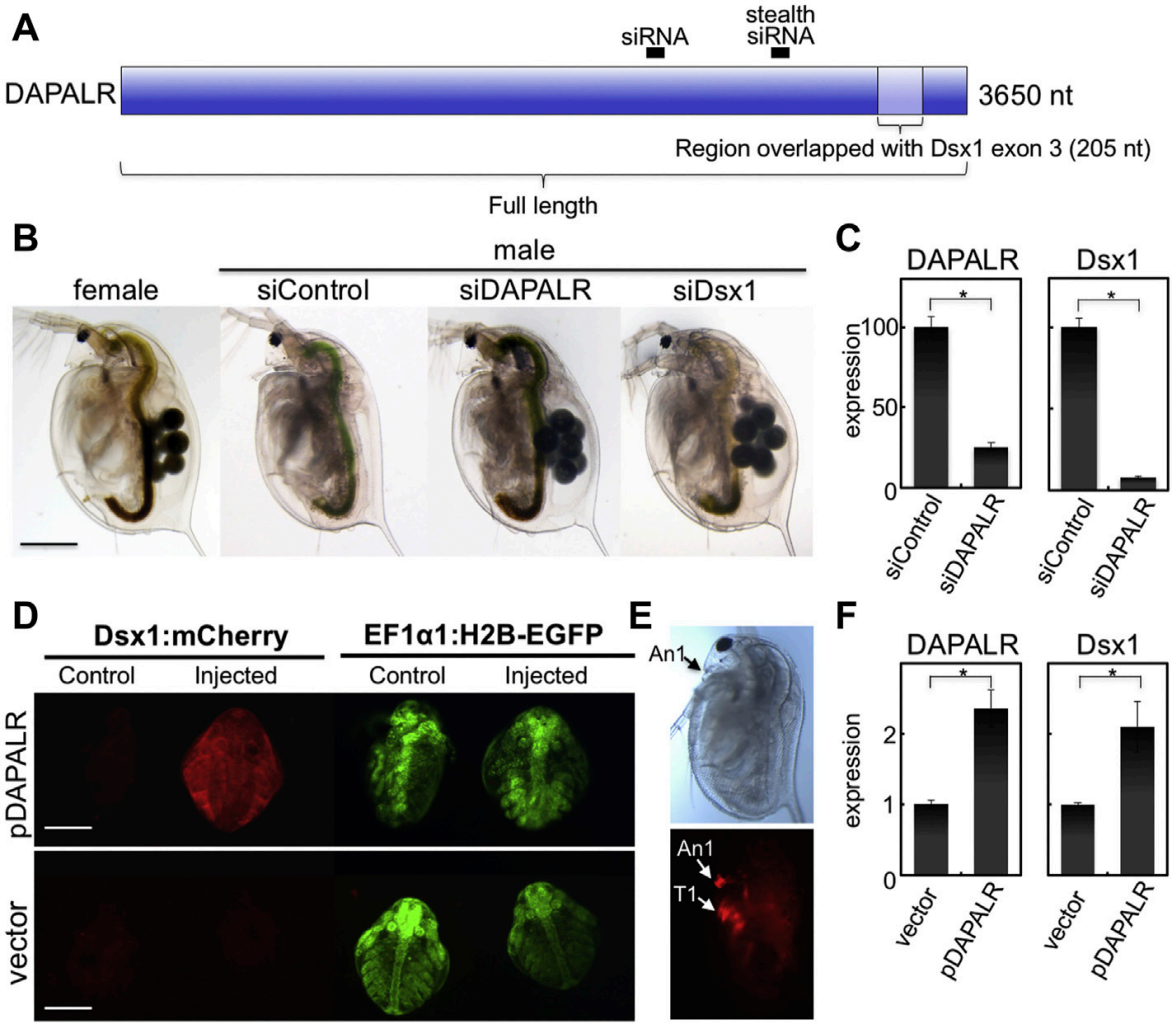
Knockdown and overexpression of *DAPALR*. **(A)** Position of small interference RNAs (siRNAs) and RNAs used for knockdown and overexpression of *DAPALR*. The siRNAs were designed in the *DAPALR*-specific region. The full-length of *DAPALR* or its region overlapped with *Dsx1* exon 3 (*Dsx1*α-isoform specific region) and was ectopically expressed in females. **(B)** Lateral view of *D. magna* injected with stealth siRNAs. *DAPALR*- and *Dsx1*-stealth siRNA-injected daphniids produced eggs in their brood chamber located in the dorsal region, in contrast to control-stealth siRNA-injected embryos. Scale bar, 500 μm. **(C)** Gene expression profile of *DAPALR* and *Dsx1* in embryos injected with stealth siRNAs of *DAPALR* (left panel) and *Dsx1* (right panel). Error bars indicate the standard error of the mean (*n* = 3). ∗*p* < 0.05 (Student’s *t* test). **(D)** Ventral view of female embryos of the *Dsx1*-reporter strain injected with *DAPALR*-expression plasmid (pDAPALR) and control plasmid (vector). **(E)** Lateral view of a female juvenile overexpressing *DAPALR*. An1, first antennae; T1, thoracic appendage 1. Scale bar, 200 μm. **(F)** Gene expression profile of *DAPALR* and *Dsx1* in embryos injected with pDAPALR (left panel) and vector (right panel). Error bars indicate the standard error of the mean (*n* = 3). ∗*p* < 0.05 (Student’s t test). Adapted from Kato et al. (2018).

*Dsx1* gene transactivation function of the *Dsx1α* 5′ UTR is attributed to the inclusion of this 5′ UTR sequence within long noncoding RNA *DAPALR* (*Dsx1* alpha promoter associated long RNA) (Figure 12A). This lncRNA is transcribed from upstream of the transcription start site of *Dsx1α* mRNA in sense orientation and overlaps *Dsx1α* 5′ UTR (Figure 3) (Kato et al., 2018). *DAPALR* is a capped and non-polyadenylated RNA. This lncRNA expression is male-specific as well as *Dsx1* but its expression level is around 10 times lower than that of *Dsx1*. *DAPALR* is activated by Vrille, which may suggest co-regulation of *DAPALR* with *Dsx1*. RNAi-mediated knockdown of *DAPALR* in males led to their feminization both in somatic tissues and germline, which resulted in the production of offspring (Figure 12B). This feminized phenotype was caused by a reduction of the *Dsx1* transcript level (Figure 12C). In contrast, overexpression of *DAPALR* in females induced upregulation of the *Dsx1* gene, resulting in the development of the male trait, elongation of the first antennae (Figures 12D–F). *DAPALR* activates *Dsx1* in *trans* and *Dsx1α* 5′ UTR functions as a transactivation element of *DAPALR* (Kato et al., 2018).

**FIGURE 13:**
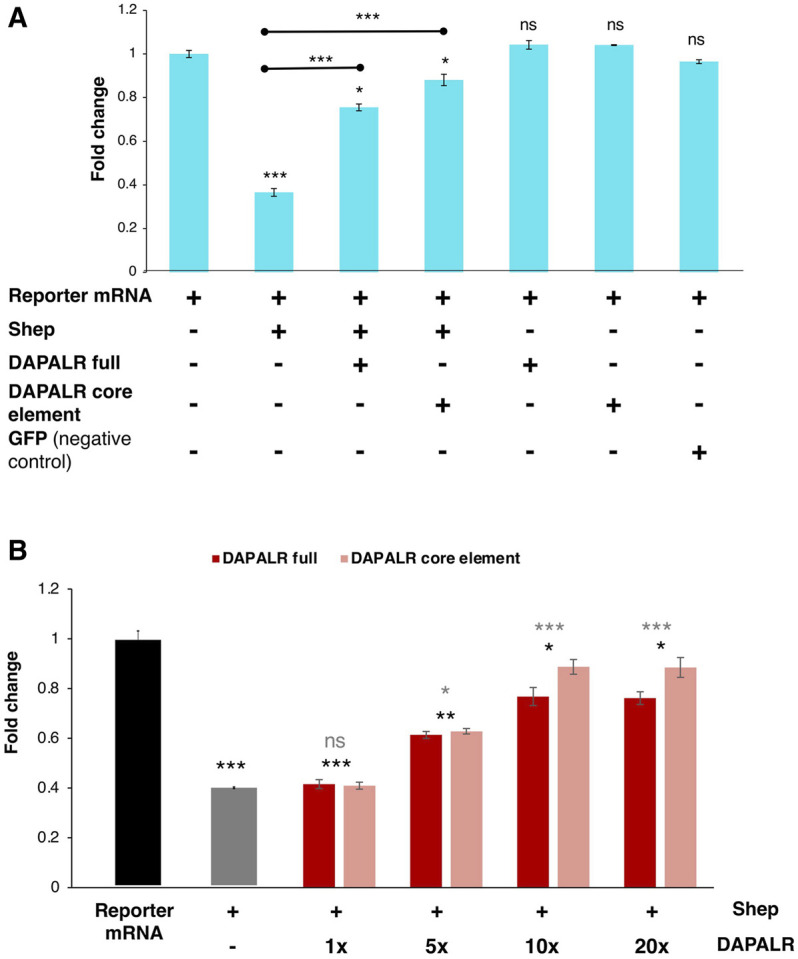
*Dsx1* post-transcription regulation by *DAPALR* and *Shep in vitro*. **(A)** Relative luciferase activity after *in vitro* translation assay of *Dsx1* 5′ UTR-Luc reporter mRNA with intact TGE upon addition of *Shep* mRNA, *DAPALR* full RNA, *DAPALR* core element, and *GFP* mRNA (negative control). Samples were compared against the expression of the *Dsx1* 5′ UTR-Luc reporter mRNA without the addition of any other mRNAs. The endpoints of the line above the bars show which samples were additionally compared statistically. **(B)** Relative luciferase activity after *in vitro* translation assay of *Dsx1* 5′ UTR-Luc reporter mRNA with *Shep* and different concentrations of full region of *DAPALR* and its core element. Error bars indicate the standard error of the mean, *n* = 3. Black asterisks show significant statistics compared with the expression of the *Dsx1* 5′ UTR-Luc reporter mRNA. Gray asterisks show significant statistics compared with the Reporter mRNA with *Shep*. Error bars indicate the standard error of the mean, *n* = 3. *p < 0.05, **p < 0.01, ***p < 0.001, ns: not significant (Student’s *t*-test). Adapted from Perez et al. (2021).

In *in vitro* translation system including the reporter mRNA harboring *Dsx1α* 5′ UTR, *DAPALR* addition canceled the Shep-dependent translational suppression (Perez et al., 2021). Similarly, *Dsx1α* 5′ UTR had the ability for canceling the Shep function (Figure 13A). Thus, *Dsx1α* 5′ UTR was also designated as a “DAPALR core element” (Figure 3). Importantly, *DAPALR* and its core element inhibited the function of Shep as a translational repressor in a dose-dependent manner (Figure 13B). *DAPALR* functions as a decoy of Shep through its core element that corresponds to *Dsx1α* 5′ UTR, which in turn increases *Dsx1* expression.

## Concluding Remarks

**FIGURE 14:**
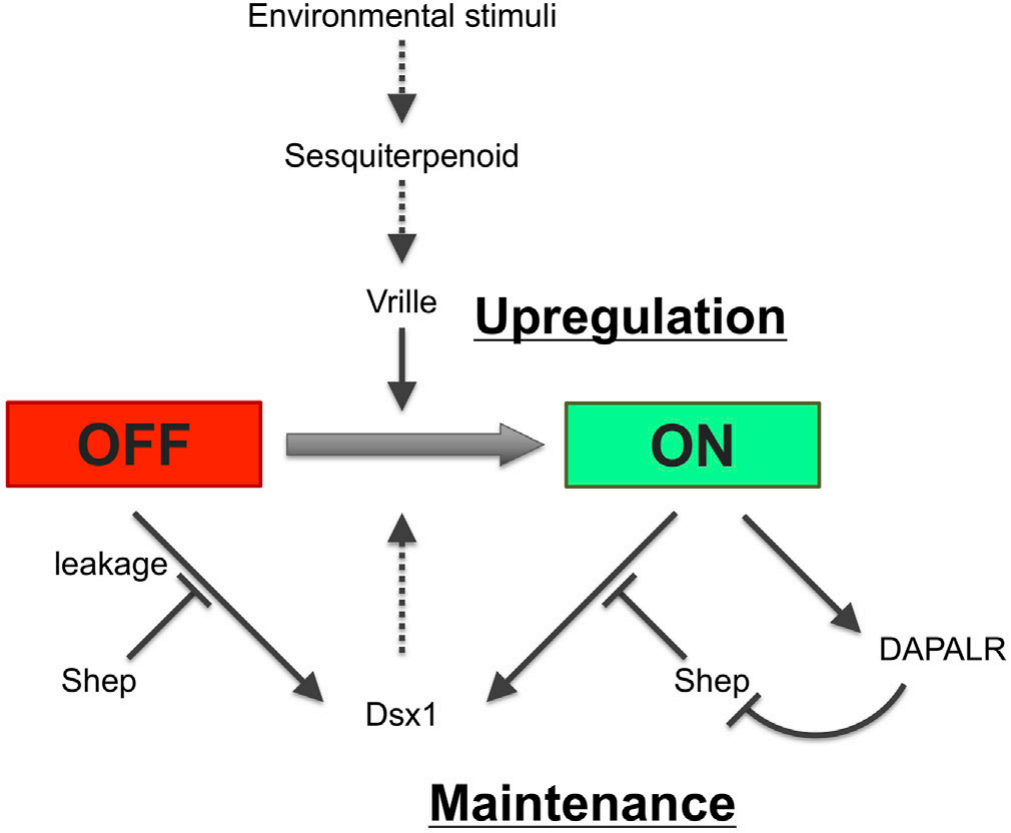
Potential regulatory network for *Dsx1* expression. Solid and dotted lines indicate validated and non-validated relationships by experiments. Figure inspiration was drawn from Huang et al. (2015).

Figure 14 shows how each regulation for *Dsx1* expression is potentially interconnected. Adult parthenogenetic females detect environmental cues and convert them to sesquiterpenoid signaling that leads to the sexual commitment of oocytes from females to males. This hormone also leads to the activation of the *Vrille* gene at the gastrulation stage. Vrille may bind to the *Dsx1* enhancer element and triggers activation of *Dsx1*, a master regulator of male development. Importantly, the active *Dsx1* locus also produces lncRNA *DAPALR* that overlaps with *Dsx1α* 5′ UTR in the same orientation. With a potential *Dsx* binding site in the *Dsx1* intron 2, *Dsx1* possibly activates its own expression *via* a positive feedback loop, which may contribute to the maintenance of *Dsx1* expression throughout development. Loss- and gain-of-function analyses of *Dsx1* and its regulators such as *Vrille*, *Shep*, and *DAPALR* provided us evidence that ectopic *Dsx1* expression in females resulted in intersex phenotypes. However, the sexual ambiguity of *Daphnia* is rare in nature, suggesting robust regulation of *Dsx1* expression. To achieve stringent suppression of *Dsx1* expression in females, Shep might be used for translational repression of unintentional *Dsx1α* mRNA by promoter leakage that may cause the autoregulation of *Dsx1*. In contrast, in males, *DAPALR* evicts Shep and unlocks *Dsx1* translation. This post-transcriptional regulation may function as a fail-safe system by which *Dsx1* expression is guaranteed and sexual ambiguity is avoided (Perez et al., 2021). It is also possible that the epigenetic state of the *Dsx1* locus may be changed from closed to opened chromatin as a memory of the environmental information that the mother has experienced.

The mechanistic bases of the unique *Dsx1* control system in the upregulation and maintenance phases during development would be an important future direction. Understanding the molecular pathway between the maternal sesquiterpenoid signaling and *Dsx1* upregulation could not only provide us evolutionary insights into the diversity of upstream components in the sex determination cascades of animals but also lead to unraveling mechanisms of transgenerational regulation of development in response to environmental stimuli. As described in this review, the masculinized cells that migrate the ventral region in early embryos might be a key cell population that lets its surrounding cells know the sexual fate. For understanding how the male-specific *Dsx1* expression is maintained, it would be important to clarify the positive-feedback regulation of *Dsx1* and the factors involved in the epigenetic regulations. To prove the decoy model of *DAPALR* and Shep, the stoichiometry of the post-transcriptional regulators and the target *Dsx1* mRNA must be investigated. For these future objectives and challenges, *in vivo* functional analysis of *Dsx1* regulators in *Daphnia* must be moved from the individual level to tissue and cellular levels. By using CRISPR/Cas or TALEN-mediated knock-in (Nakanishi et al., 2015; Nakanishi et al., 2016; Kumagai et al., 2017), specific cell population needs to be fluorescently labeled, isolated, and subjected to single-cell analysis. This approach will allow us to move away from current analyses using heterogeneous cell populations and to identify *Dsx1* regulators that control developmental stage-, tissue- and cell-specific traits at high resolution, which provides us a comprehensive understanding of *Dsx1* regulation for environmental sex determination in *D. magna*.
